# Supplementary figures and images for: Nuclear α-Synuclein-Derived Cytotoxic Effect via Altered Ribosomal RNA Processing in Primary Mouse Embryonic Fibroblasts
Source: Int J Mol Sci. 2023 Jan 21;24(3):2132. doi: 10.3390/ijms24032132 (PMC9917353; doi:10.3390/ijms24032132)

# Supplementary Figure S1

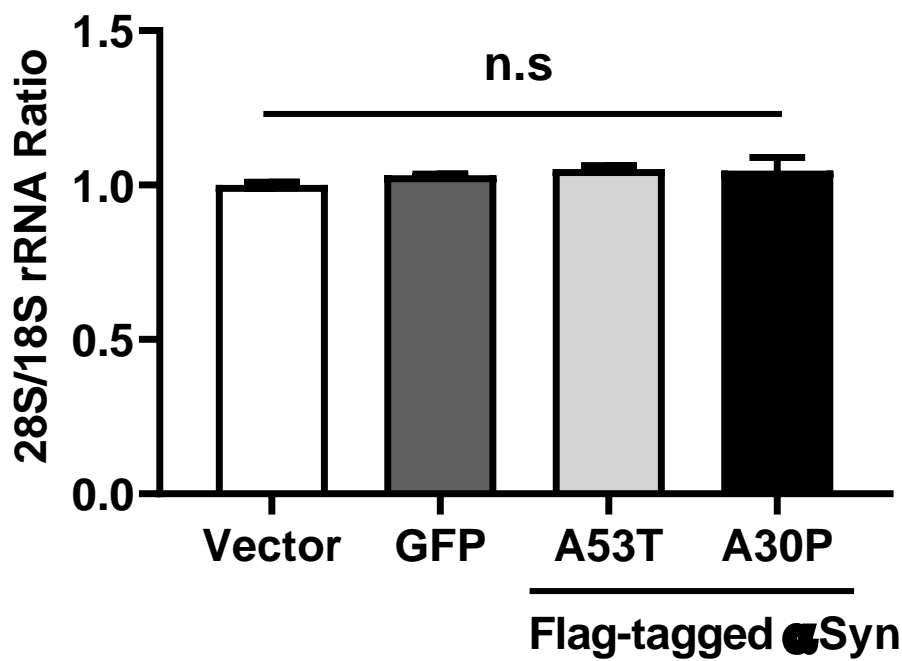

# Supplementary Figure S2

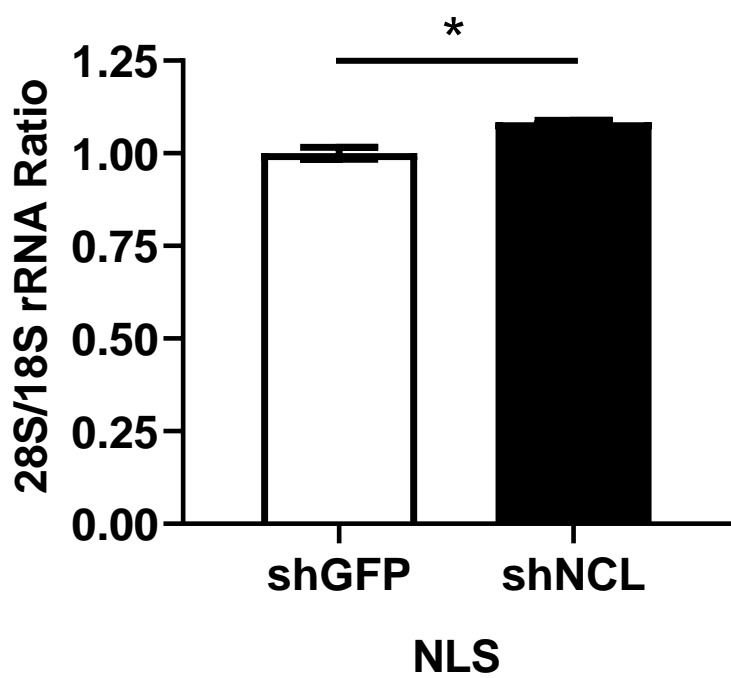

Supplement: Supplementary file 1 [file ijms-24-02132-s001.zip › ijms-2154794-supplementary.pdf]
